# Human herpesvirus-8 infection in Tunisian adult acute leukemia patients

**DOI:** 10.4314/ahs.v23i1.52

**Published:** 2023-03

**Authors:** Imene Handous, Naila Hannachi, Bechir Achour, Manel Marzouk, Olfa Hazgui, Saloua Yacoub, Abderrahim Khelif, Jalel Boukadida

**Affiliations:** 1 Université de Sfax, Ecole nationale d'Ingénieurs de Sfax, 3038, Sfax, Tunisie; 2 Université de Sousse, Faculté de Médecine de Sousse, Laboratoire de Microbiologie, UR12SP34, Hôpital Farhat Hached,4000, Sousse,Tunisie; 3 Université de Sousse, Faculté de Médecine de Sousse, Service d'Hématologie, Hôpital Farhat Hached, 4000, Sousse, Tunisie; 4 Centre Régional de Transfusion Sanguine, Hôpital Farhat Hached,4000, Sousse,Tunisie

**Keywords:** Human herpesvirus 8, acute leukemia patients, blood donors, prevalence

## Abstract

**Background:**

Human herpesvirus 8 (HHV-8) has been linked to the development of Kaposi's sarcoma (KS)and multiple other hematologic malignant disorders. However, the role of HHV-8 in acute leukemia patients is unknown.

**Objectives:**

The objective of this study was to determine the prevalence of HHV-8 in Tunisian acute leukemia patients and in healthy blood donors.

**Methods:**

An indirect immunofluorescence test was used to detect the presence of anti-HHV8 antibodies. Nested PCR was used for the detection of HHV-8 DNAemia in samples of plasma.

**Results:**

The seroprevalence of HHV-8 was significantly higher in acute leukemia patients (21,4% ,15/70) than in healthy blood donors (7,1%, 5/70), (p= 0.02). Gender, type of disease, status of disease, prior blood transfusion, and outcome were not associated with HHV-8 seroprevalence. However, among acute leukemia patients, HHV-8 seroprevalence was statistically associated with older age > 40 years of age, (p=0.002). HHV-8 DNAemia was detected (1,4%) in only one patient of acute myeloid leukemia (AML) and none of the healthy blood donors.

**Conclusions:**

The seroprevalence of HHV-8 infection in Tunisian adult acute leukemia patients was three times as high compared to healthy blood donors, suggesting that patients with acute leukemia might be at increased risk of HHV-8 infection.

## Introduction

Kaposi's sarcoma-associated herpesvirus (KSHV) also known as Human herpesvirus-8 (HHV-8) is considered as the etilogic agent of all five types of Kaposi's sarcoma: classic Mediterranean, African endemic, iatrogenic in immunocompromised patients, acquired immune deficiency syndrome (AIDS)-related epidemic and non-epidemic KS^1^, ^2^. HHV-8 has been implicated also in a number of malignant disorders including pleural effusion lymphoma, multicentric Castleman's disease and some lymphoproliferative diseases 3, 4. HHV-8, unlike other human herpesviruses, is not widely distributed^5^. HHV-8 seroprevalence varies geographically and between sub-populations^6^,^7^. Based on HHV8 seropositivity, the world has been divided into three regions: those with less than 5% seroprevalence, such as North America and Northern Europe; those with intermediate seroprevalence, ranging from 5% to 20% in Mediterranean and Middle Eastern countries, to more than 50% in many African populations and regions, such as Sudan. 8–12.HHV-8 infection does not appear to be widespread in the Tunisian community, and the observed seroprevalence of anti-HHV-8 in apparently healthy persons was 13.8 %, compared to 17-40 % in patients having kidney renal transplants^10^, ^13^. HHV-8 is spread from one person mainly through saliva, but infection can also occur through sexual contact^14^, blood transfusion^15^ and by solid organ transplantation (SOT)^16^. HHV-8 infections are usually asymptomatic in immunocompetent persons. HHV-8 may reactivate and result in symptomatic infections, and may cause even fatal complication, especially in the context of immunodeficiency and immunosuppression. While multiple studies of HHV-8 infection have been reported an increased rate of HHV-8 infection, especially in the setting of organ transplant recipients, HHV-8 infection is uncommon in the allo-HSCT recipients^17^, and little information about HHV-8 infection in non-transplanted patients with acute leukemia undergoing chemotherapy is available. This study examined at the occurrence of HHV-8 infection in acute leukemia patients and in blood donors from central east of Tunisia.

## Methods

### Study design and participants

In this cross-sectional study, 70 acute leukemia patients (19 females and 51 males) and 70 age and gender-matched blood donors were included between January 2016 and December 2018. These patients were diagnosed and treated with conventional chemotherapy at Farhat Hached University Hospital's Department of Hematology. All the included patients had no previously a history of cancer ,hematological, or immunological illness, and no sexual transmitted diseases (human immunodeficiency virus, hepatitis B or C). None of these newly diagnosed leukaemia patients received allogeneic or autologous hematopoietic stem cell transplantation.

The current study was conducted in line with Helsinki Declaration principles and was authorized by the Ethics Committee and Medical research of the Farhat Hached University Hospital in Sousse, Tunisia (Reference number IRB00008931). Before beginning the research, all participants provided written informed consent.

### Serological analysis

A commercially available indirect immunofluorescence assay (IFA) was used to identify anti-HHV-8 lytic IgG antibodies in serum samples (Kit Biotrin HHV-8-IgG-IFA, Dublin, Ireland).

According to the manufacturer's catalogue, the sensitivity and specificity of the present immunofluorescence test were 100% and 94%, respectively, making them appropriate for HHV-8 screening.

### HHV-8 PCR detection

HHV-8 DNA was extracted from a 200-µL of plasma using a QIAamp DNA Mini Kit (QIAGEN, Germany) and DNA was eluted in a final volume of 50 µL.

The detection of HHV-8 DNA was performed by a highly sensitive and specific nested PCR as previously described^18^. Ten microliter of these extracted DNA samples was used as template for the nested amplification. The first round PCR used primers from ORF26, to amplify 233-bp product. The primers were KS1 5′AGCCGAAAGGATTCCACCAT 3″, and KS2 5′TCCGTGTTGTCTACGTCCAG3″ and for second PCR reaction KS3 5′ TATTCTGCAGCAGCTGTTGG and KS4 5′ TCTACGTCCAGACGATATGTGC 3′. The Positive second PCR reactions generated 138 pb amplicons for ORF 26. PCR were performed with appropriate negative controls (DNA extracted from donor plasma samples, with negative HHV-8 IgG Immunofluorescent assays) and positive controls (Kaposi's sarcoma samples) in each reaction. The PCR products were analysed by electrophoresis on a 2% agarose gel containing ethidium bromide, and were visualized under ultraviolet light alongside with the 100 bp DNA ladder.

### Statistical analysis

SPSS statistics software (version 20.0) was used for statistical analysis. To compare variables, the Chi-square test or Fisher's exact test was employed. Data were provided as mean±SD and when indicated as an absolute number and percentage. If the p-value is lower than 0.05, the association was considered statistically significant.

## Results

This study included 70 patients with acute leukemia (38 acute lymphoblastic leukemia (ALL) and 32 acute myeloid leukemia (AML)). The patient ages varied from 19 to 64 years, with a mean of 33,22±13.4 years. Table 1 summarizes the detection rate of anti-HHV-8-IgG and DNA detection rates. HHV-8 seropositivity in acute leukemia patients was significantly higher than in the control group of healthy blood donors, (21.4% (15/70) vs 7.1% (5/70), p=0.02).

**Table 1:** Detection of HHV-8 antibodies and DNA

Groups			
	Acute leukemia N=70 No. (%)	Blood donors N=70 No. (%)	P value
Mean age, years	33.2(19–64)	32.5(18–60)	> 0.05
Sex, male/female	51/19	51/19	> 0.05
**Anti-HHV-8 IgG**			
Positive	15(21.4)	5 (7.1)	0.02
Negative	55(78.6)	65(92.9)	
**HHV-8 DNA**			
Positive	1(1.4)	0(0)	
Negative	69(98.6)	70 (100)	> 0.05

Chi-Square test was used for statistical analysis and p<0.05 was considered statistically significant

The association between demographic and clinical characteristics of leukemia patients with positive and negative anti-HHV-8-IgG antibodies is shown in Table 2.

**Table 2:** The relationship between demographic and clinical characteristics of patients with positive anti-HHV-8 and negative anti-HHV8

		Acute leukemia		
			
		Positive anti-HHV-8	Negative anti-HHV-8	P value

	N=70	N=15	N=55	
Characteristic	No. (%)	No. (%)	No. (%)	
**Gender**				
Female	19 (27.1)	2(13.3)	17 (30.9)	0.21
Male	51 (72.9)	13 (86.7)	38 (69.1)	
**Age group (years)**				
19–40	39 (55.7)	3 (20)	36 (65.5)	0.002
> 40	31 (44.3)	12 (80)	19 (34.5)	
**Type of disease**				
AML	32 (45.7)	6 (40)	26 (47.3)	0.771
ALL	38 (54.3)	9 (60)	29 (52.7)	
**Status of disease**				
Newly diagnosed	49 (70)	10 (66.7)	39 (70.9)	0.463
Remission	8 (11.4)	3 (20)	5 (9)	
Relapse/ refractory	13 (18.6)	2 (13.3)	11 (20)	
**Prior blood transfusion**				
**Yes**	29 (41.4)	8 (53.3)	21(38.2)	0.378
**No**	41(58.6)	7 (46.6)	34(61.8)	
**Outcome**				
**Alive**	48 (68.6)	9 (60)	39 (70.9)	0.619
**Dead**	22 (31.4)	6 (40)	16 (29.1)	

Chi-Square test was used for statistical analysis and p<0.05 was considered statistically significant

The seropositivity rate did not differ statistically based on gender, type of disease, status of disease, prior blood transfusion and outcome. Interestingly, the seropositivity for HHV-8 was shown to be substantially related with being over 40 years old (p=0.002).

HHV-8 DNA was detectable in only one patient, whereas none of healthy blood donors showed positive for HHV-8 DNAemia. The patient was a 49-year-old man with AML, presented fever, severe pancytopenia and pneumonia, but might be mistaken for a several viral infections. The patient died few days after induction therapy.

## Discussion

Epidemiological data of HHV-8 in acute leukemia patients is poor worldwide. Studies of prevalence of HHV-8 in those patients, especially those from Mediterranean Northern Africa, have not, to date, been reported. This is the first analysis of the prevalence of HHV-8 infection in patients with acute leukemia from central east of Tunisia.

The seroprevalence rate of HHV-8 infection among healthy blood donors was relatively lower (7.1%) than with previous studies in Tunisia reporting (13-15%) in blood donors^13^,^19^, 13% in pregnant women, and 12% in children^13^, which suggests that Tunisia is classified into the intermediate prevalence region, although large geographic variation seems to exist within the Mediterranean regions 20. In this study, none of the healthy blood donors showed evidence of HHV-8 DNAemia. This is consistent with several studies that could not find HHV-8 DNAemia in seropositive blood donors from regions with low HHV-8 prevalence^21^,^22^. In contrast, strong evidence that HHV-8 is transmitted by blood transfusion has been reported previously 15, 23, 24.

Blood unit screening might be considered in countries with moderate to high HHV-8 seroprevalence, particularly when destined for immunocompromised patients 15, 22. Since the prevalence of HHV-8 is low, our results do not support the routine blood banks screening for HHV-8^13^.

Immunosuppression has been identified as a key factor in the development of HHV-8 infection 20, 25, 26. In this study, acute leukemia patients had an increased seroprevalence of HHV-8 than healthy blood donors (21.4% vs 7.1%, p=0.02) suggesting that they are more likely to become infected with HHV-8 than immunocompetent individuals. Similar to our study, in Taiwanese patients with hematological disorders including lymphoma, leukemia, autoimmune cytopenias and myeloproliferative disorders; higher seropositive rate was observed, compared to the overall population (24.5% vs. 10.5%, p = 0.001)^26^. In contrast to another study, the seropositivity of HHV-8 in African cancer patients was not substantially different from the overall seropositivity among blood donors 27.

This difference might be due to a variety of factors, including changes in methods, technique sensitivity, geographical location, and subgroups.

Similar to previous reports on patients with hematological diseases, we did not find any correlation between the seropositive rate and gender^26^. Yet, HHV-8 seropositivity was associated with higher age (more than 40 years) in adults patients.

This can be due to an ongoing transmission among adults. This finding is consistent with the findings of a prior investigation that found an increasing prevalence of HHV-8 in cancer patients aged 50 years or older from China 28. These findings are consistent also with previous studies for Mediterranean and African countries where the seroprevalence of HHV-8 were age dependent^20^,^29^,^30^.

HHV-8 DNA have been linked to a various illness, although the virus's involvement in some of these diseases is mostly unknown as much as KS preceding SOT^14^,^31^. In SOT^32^ and HIV-infected subjects^33^, the risk of KS is increased (up to 200-fold) while it is low in allo-HSCT^34^. HHV-8 is an oncogene virus, as evidenced by its presence in human malignancies, by the in vitro transforming properties of several of its viral genes, and other cofactors such as immunosuppressive cytokines which are involved in the progression of angioproliferative and inflammatory KS lesions^35^.

In our study, only one of acute leukemia patients who screened positive for anti-HHV-8 IgG was found to harbor also HHV-8 DNA in plasma. Our findings suggest that the detection of HHV-8 DNAemia in Tunisian acute leukemia patients is not common. Similar findings were reported in Iranian patients with HM, where HHV-8 DNAemia were found in 4 (6.5%) patients with HM which included one (3.7%) patient with AML, 3 (13.6%) chronic myeloid leukemia and none was found in patient with ALL and lymphoma. In contrast, Hen and his collaborators have reported a higher prevalence of HHV-8 DNA in 10.29% of Taiwanese leukemia patients in peripheral blood mononuclear cells^36^. Although, HHV-8 has been linked with several lymphoproliferative disorders^4^,^26^, but whether HHV-8 is involved in acute leukemia patients remains unclear. The presence of HHV-8 DNA may also be due to reactivation of latent virus especially in immunocompromised patients.

No association was noted between HHV-8 seropositivity and acute leukemia status. Thus, if leukemia status was associated with greater HHV-8 infection, we don't know whether tumorigenesis preceded or followed HHV-8 infection. To establish the temporal association between acute leukemia status and HHV-8 infection, a prospective study design is required^28^.

Herpesvirus infections are frequently associated with increased morbidity and death rates in transfused patients^37^. In this study, no statistically significant association was found between HHV-8 infection in transfused and non-transfused patients with acute leukemia. In contrast, HHV-8 transmission through blood transfusion has been reported in Uganda, a country with a high prevalence of classical KS, where HHV-8 seropositivity was shown to be significantly more frequent in transfused versus never-transfused children with sickle-cell disease^38^.

While the mode of transmission is unknown, multiple transfusions do not appear to affect HHV-8 infection in this study. More research is needed to better understand the possible risk of HHV-8 infection in acute leukemia patients via whole blood or blood derivatives. Environmental or social factors are likely to have a role in increasing HHV-8 seroprevalence in acute leukemia patients. HHV-8 is transmitted through close sexual contact and saliva, but infection may also be acquired by sharing of utensils since it can contain significant amounts of HHV-8,^28^,^39^,^40^.

The exact higher risk practices that enhance the risk for HHV-8 transmission have not yet fully been defined in the different patient subgroups and regions 41. Only few previous studies have looked into prevalence and modes of transmission, especially in acute leukemia patients. But further research is required in this direction.

This study had some limitations: to predict a more realistic incidence of HHV-8 infection research cohorts should be in the overall population rather than in blood donors. Blood donors are not considered with high-risk behaviors. The lack of detectable HHV-8 DNA in these patients may be a result of limited sample size. As a result, longitudinal studies of HHV-8 in a larger number of individuals with acute leukemia are needed. Although, the nested PCR technique used in the current study has a higher sensitivity when compared to the other conventional PCR techniques adopted in other past studies^22^. PCR results must be evaluated in combination with clinical and histological evidence, and they cannot be used to determine a diagnosis on their own. Furthermore, PCR results are frequently negative in remission patients or asymptomatic HHV-8 carriers^22^, ^42^.

In conclusion, our results showed that Tunisian patients with acute leukemia are at increased risk of HHV-8 infection and that HHV-8 infection is more common in elderly patients (age > 40). More research is needed to better define co-factors associated with HHV-8 infection in these patient groups to define effective prevention and treatment strategies.
